# Terahertz meets sculptural and architectural art: Evaluation and conservation of stone objects with T-ray technology

**DOI:** 10.1038/srep14842

**Published:** 2015-10-07

**Authors:** K. Krügener, M. Schwerdtfeger, S. F. Busch, A. Soltani, E. Castro-Camus, M. Koch, W. Viöl

**Affiliations:** 1 University of Applied Sciences and Arts, Faculty of Natural Sciences and Technology, Von-Ossietzky-Str. 99, Göttingen 37085, Germany; 2 Philipps-Universität Marburg, Department of Physics, Renthof 5, Marburg 35032, Germany; 3 Centro de Investigaciones en Optica A.C., Loma del Bosque 115, Lomas del Campestre, Leon, Guanajuato 37150, Mexico; 4 Fraunhofer Application Center for Plasma and Photonics, Von-Ossietzky-Str. 100, Göttingen 37085, Germany

## Abstract

Conservation of cultural heritage is an area where novel scientific techniques are having enormous impact. Given the value and uniqueness of art pieces, non-invasive diagnostic methods are highly appreciated by conservators. Terahertz radiation has shown enormous potential as non-contact probe that can be used for the three-dimensional reconstruction of internal structure of stone-made objects. In this article we report the evaluation of the internal damage state of two art pieces, a medallion from the Castle of Celle and a window sill from the St. Peter of Trier Cathedral. We also used terahertz radiation to follow and assess the restoration process of the window sill. We found that terahertz spectroscopy is an excellent non-destructive evaluation method for stone artwork that shows enormous potential as a tool for conservation.

The study of cultural heritage and the development of cutting edge scientific techniques have traditionally been academic fields with relatively little interaction. Yet, in recent years both scientists and researchers in the conservation and restoration area have seen the enormous potential of combining their expertise in order to, either, restore culturally valuable objects, or reveal information about them that was not accessible before^1^,^2^,^3^. Terahertz time-domain spectroscopy is a technology that emerged about three decades ago and that has seen considerable progress since then^4^,^5^. Although expensive, terahertz spectroscopy systems are now commercially available^6^,^7^, and interesting variations of that technique that could significantly lower the cost of such systems are being explored^8^. Terahertz radiation is non-ionizing and allows seeing through many materials used in the fabrication of culturally valuable objects, such as paper manuscripts^9^, stones^10^, clay^11^ and wood^12^,^13^ among many others^14^,^15^,^16^,^17^,^18^,^19^,^20^. It has also been demonstrated that it is possible to reconstruct three-dimensional air-gaps or material structures within those objects which could be an invaluable tool for the evaluation of damage and restoration of paintings, pottery, sculptures, buildings, etc.^10^ Damage of environmentally exposed stone objects is often externally invisible. Temperature and humidity fluctuations, particularly when frost or saline accumulation forms can result in the generation of cracks, gaps, and even full detachment of parts of a sculptural and architectural artwork. In terms of other techniques, portable X-ray machines are relatively economical, however, they involve radiological hazards which are, in general, incompatible with the study of buildings, unless the buildings can be vacated. In addition they provide only images with two-dimensional information. Infrared thermography also provides two-dimensional information and in many cases requires the object to be heated up, which is not always possible or desirable. X-ray tomography does provide three-dimensional information, however, it is only applicable to small pieces that can be taken to the tomograph, but not to attached building fragments.

In this article we present terahertz spectroscopy as a useful non-destructive tool to analyse the structural damage of stone-made objects and present the application of this technique to two original artwork pieces. In particular we aim to find cracks caused by delamination within the two study cases and evaluate the restoration process of one of them. The first sample is a stone medallion from the collection of the Lower Saxony State Museum at Hannover, and the second is a section of a window sill from the historical cloister of the Cathedral of Trier.

About the pieces studied; The first one is a medallion made of yellow lime sandstone with fragments of polychrome colouring which probably was added sometime after its creation (Fig. 1b) The former integral relief was created as one of several expressive head portrait medallions during the first half of 16th century by Levin Storch as architectural elements of the gatehouse belonging to the Castle of Celle in northern Germany. After part of the castle was damaged during the Second World War the elements of the historical-artistic ensemble were relocated^21^. Nowadays the medallion under investigation is one of six pieces (see Fig. 1a) that belong to the collection of the Lower Saxony State Museum in Hannover. The medallion studied here is currently not exhibited given that it is under restoration and is kept at the storage room of the museum. It consists of several fragments, glued in previous restorations for the protection and preservation after getting heavily damaged by fire, water and frost. The medallion in its current state shows damage by shell formation in several parts. In preparation for the terahertz study we performed a quick selection of the area to be studied by conventional knocking method (percussion with a metal). Using Fig. 2e as a reference, at point a and c there is a clear sound suggesting delamination under the surface. At point b no sound was perceptible but we suspected that the delimitation went up to this point. At point d no sound could be heard, we choose this point as a reference because there was clearly solid stone material. The dimensions of the medallion are: length 58 cm, width 57 cm and height 9.5 cm.

The second object we studied is a fragment of a window sill from the St. Peter of Trier Cathedral which is the oldest episcopal church in Germany and has been recognised since 1986 as part of the UNESCO world heritage of Roman historical architectural monuments (see Fig. 1c). The cathedral was built primarily from Kordeler sandstone. Kordeler sandstone, also known as Voltziensandstein, is a yellowish-grey or red sandstone mined in Kordel close to Trier in Rhineland-Palatinate, Germany. Kordeler sandstone is an important cultural rock used for many historical buildings in the region around Trier since Roman times. It is a stone that shows good weather resistance. However, typical signs of surface damage caused by weather for this stone include sanding and formation of shells. Such surface damage can lead to cavities and finally to flaking of the surface^22^. Original stones or parts of architectural elements of many historically valuable buildings sometimes need to be replaced during conservation measures because of their degree of deterioration. This was the case with fragments of the window sill of the suffragan bishop chapel during a restoration in 2013. The piece dates from the 13th century and is early gothic style. Figure 1d shows a cut fragment with the typical damages of shell formation we analysed during our research.

## Results

Terahertz time-domain spectroscopy is a peculiar spectroscopic technique. Instead of recording the light intensity as function of its wavelength (or color), usually done in traditional spectroscopy, very short single-cycle pulses of radiation are produced and their time-dependent waveform is recorded. This allows using the terahertz pulses to find objects that they interact with as they propagate. This is possible because the surface of each object found in the pulse’s path will generate a small echo. The time taken for each echo to propagate back from each object to the receiver can be associated to the position of the object^23^. In this case we used this technique to find internal cracks or air-gaps within the objects described in the section before produced by delamination of the stone. Further details about the terahertz technique and the data analysis can be found in the Methods section. It is worth mentioning that various historically relevant stones have been characterized in the terahertz band before^10^.

### The medallion

The first experiment that we are presenting is the evaluation of the medallion described in the previous section which was part of the Castle of Celle. Terahertz reflection pulses were recorded in various points across the medallion. Figure 2(a–d) shows a series of time domain data sets that correspond to the pulses reflected at some selected positions, marked in Fig. 2e. It is possible to see that points (a), (b) and (c) show small, but distinguishable echoes in the 35 ps to 45 ps window. This means that an air-gap buried approximately 2.3 mm to 3.4 mm underneath the surface at those positions. Similarly the absence of an echo at point (d) indicates that the stone is solid in that position. Additional points were measured in the area and are not shown for clarity of the figure. From the analysis of all the points measured it is possible to approximately delimit the damaged area (shown in the figure with dashed lines).

### The window sill

**Figure 1:**
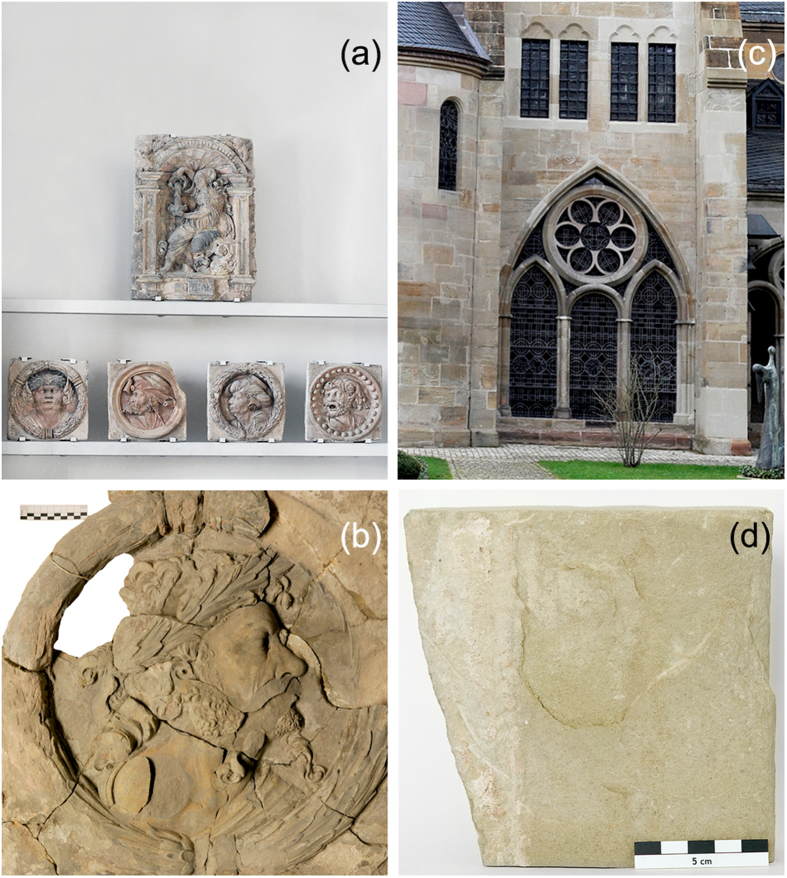
(**a**) Exhibit of the Lower Saxony State Museum Hannover showing several stone medallions from the Castle of Celle. (**b**) Close-up photograph of the medallion under study, a length scale is shown as a reference on the upper left hand side that corresponds to 10 cm. (**c**) Image of part of the cloister of the St.Peter of Trier Cathedral. The second sample studied in this article corresponds to a fragment taken from the window sill under the third and fourth windows (from left to right) on the upper level which was replaced with a new window sill during a restoration in 2013. (**d**) Fragment of the window sill removed from the Cathedral, some damage is visible, in particular a delamination on the central part, a length scale is shown as a reference on the lower right hand side that corresponds to 5 cm. (Photographs taken by K. Krügener).

**Figure 2:**
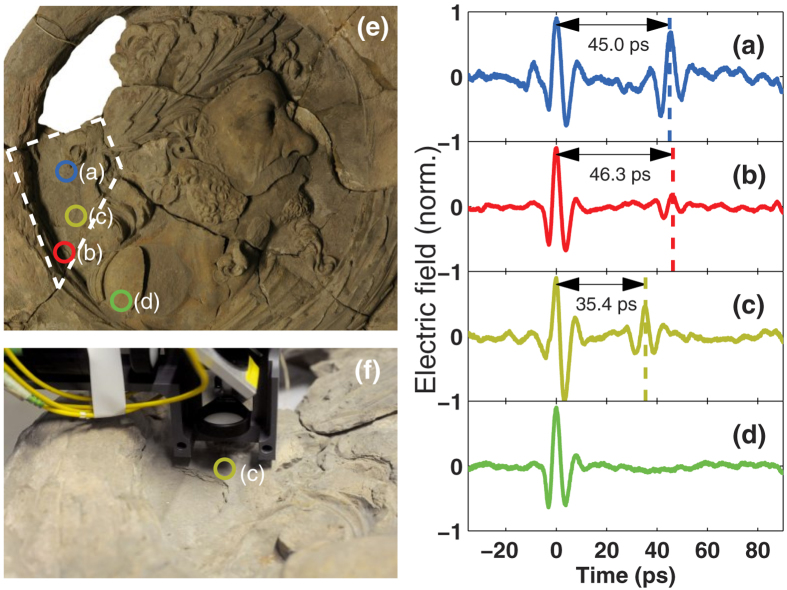
Panels (a–d) are the reflected terahertz pulses from various locations on the medallion, the positions that correspond to each measurement are marked on panel (e) It is easy to see that the area where points (a–c) lay presents an internal air gap that generates an echo 35 ps to 45 ps from the surface, which corresponds to 2.3 mm to 3.4 mm from the surface. The measurement at point (**d**) shows that the stone is solid and has no internal structure at that position, a dashed line approximately delimits the damaged area. (**f**) Photograph of the measurement process at position (**c**), the measurement point is indicated. (Photographs taken by K. Krügener).

**Figure 3:**
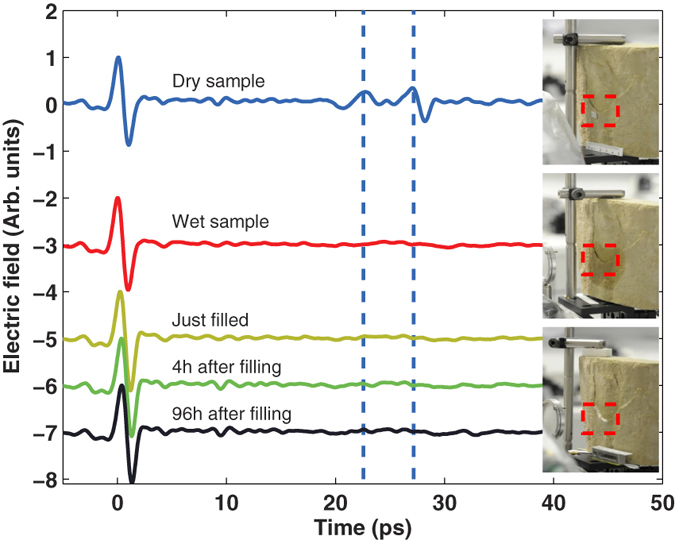
The curves show the evaluation of the repair process of the window fragment from the St. Peter of Trier Cathedral. The insets on the right hand side are photographs taken during the corresponding conservation steps and the measurement area is indicated by a red rectangle. The top curve is a terahertz pulse recorded at the position marked by a red rectangle on the inset (where the delamination is visible). The two echoes present between 20 ps and 30 ps correspond to the stone-air and air-stone interfaces of the air gap produced by the delamination, their temporal separation (0.43 ps) corresponds to an air-gap of ~0.69 mm. The echoes are no longer visible on the wet and just filled sample because water is very opaque in the terahertz band which prevents radiation from reaching the air gap, and therefore, the generation of echoes. The absence of the echoes on the pulses recorded 4 and 96 hours after the air gap was filled demonstrate that the filling was successful and that, therefore, the connection and preservation of the valuable original surface is thus achieved. (Photographs taken by K. Krügener).

**Figure 4:**
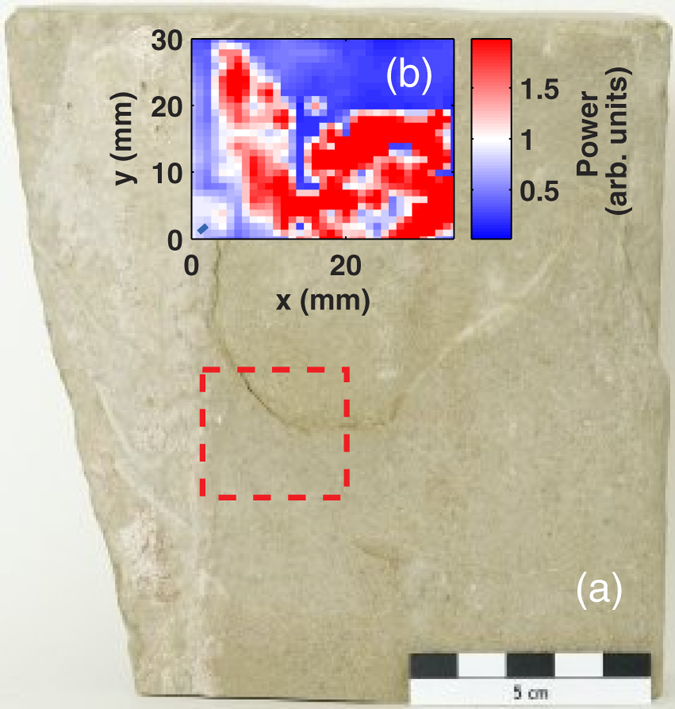
(**a**) Photograph of the window sill under investigation, the rectangle marked with dashed lines indicates the area where a terahertz reflection image was taken. (**b**) Colormap representation of the reflected THz power over the time window t > 10 ps after the first pulse. This power corresponds to the total intensity reflected by the air-gap inside the stone. The white-red area is the actual shape of the air-gap. With this image it is possible to estimate that the footprint of the air-gap which is approximately 690 mm^2^. (Photograph taken by K. Krügener).

The second sample we studied was the fragment of a window sill replaced at the Trier Cathedral in a recent restoration. This piece was also described in the previous section. The fact that this particular piece showed environmental deterioration and needed to be completely replaced allowed us to examine it in great detail in the laboratory and to test the potential of terahertz radiation in the evaluation of its restoration process as well. As seen in Fig. 1d the piece shows delamination in the central part. A ~0.7 mm thick air-gap located ~1.25 mm underneath the surface is clearly visible in the photograph. A terahertz time-domain pulse was recorded (top curve of Fig. 3). In the time-domain trace two echoes are clearly visible ~22.6 ps and ~27.2 ps after the first reflection. These two echoes correspond to the stone-air and air-stone interfaces of the air-gap. The time difference between the two pulses allows estimating that the thickness of the air-gap is approximately 0.69 mm. The window sill fragment was mounted on a two-axis motorised stage and a collection of terahertz measurements were performed on a two-dimensional mesh of points across the area indicated by a dashed rectangle on Fig. 4a. The power reflected from the sample for delays >10 ps after the first reflection was calculated for each measurement and the resulting colormap image is shown on Fig. 4b. The white-red area is an image of the air-gap. This image provides us with information on the shape and size of the gap. This information allows a better evaluation of the damage of the piece and therefore a better planning of the course of action for restoration. From the colormap it is possible to estimate that the footprint area of the air-gap is approximately 690 mm^2^. Combined with the thickness obtained from the separation between echoes it allows us to estimate that the total volume of the air cavity is 476 mm^3^. The uncertainty of this particular estimation is ±176 mm^3^ given that the pixel-to-pixel distance in the image is 2 mm. Yet the uncertainty of the instrument is dictated by the diffraction limit for the lateral (X and Y) directions (ie. ~300 *μ*m/2) and is about 80 *μ*m for the depth (Z) direction^10^. This means that the instrument could resolve the volume of this cavity with an inherent uncertainty of ±60 mm^3^.

Once the evaluation of the damaged area was performed we decided to perform a real-time follow up of the repair process. Water was injected into the gap of the stone and a second terahertz measurement was performed. In this case no echoes were visible any more (second curve of Fig. 3). This is because water is highly opaque in the terahertz band, which prevents the pulse to reach the gap and, consequently, to reflect back. Immediately after addition of water the air-gap, it was filled with Jahn M30 micro backfill grout and an additional terahertz measurement was performed (labeled just filled in Fig. 3). Once again no echoes are present. Their absence can be explained because the stone was still wet, preventing the terahertz pulse to penetrate beyond the surface. Further measurements were taken 4 and 96 hours after the filling process. Over that period the stone became dry again but the echoes in the 20 ps to 30 ps window did not reappear. This demonstrates that the air-gap was successfully filled and is consistent with a relatively small refractive index mismatch between the stone and the grout. We separately determined the refractive indices of the stone (n = 1.97) and grout (n = 2.01) using the method described in ref. 10. As a reference, we performed a test before filling the sample by just wetting it and letting it dry. The echoes were visible again in the terahertz trace after about 1.5 hours.

## Conclusions

In this article we used terahertz time-domain spectroscopy in reflection geometry as a tool for the evaluation of damage of two real stone artwork pieces. We were able to find hidden air-gaps caused by environmental deterioration. On the first sample, a 16th century stone medallion, a hidden crack 5 mm to 7 mm underneath the surface was discovered in a section of the stone. As for the second sample, a fragment of a 13th century stone window sill, terahertz imaging allowed finding the shape and dimensions of an air gap caused by delamination of the stone which is only visible on one edge. Such geometrical information was used in order to better plan the restoration of the piece. Furthermore, the conservation process of the window sill was followed step-by-step with this technique. The restoration consisted on the filling of the air-gap with a special grout. We were able to assess that the filling was successful and that no air-gap was present once the process finished. We demonstrated that terahertz spectroscopy and imaging can provide very useful information for the evaluation of damage of stone made artwork. Single point measurements by this technique can be used for a “quick” qualitative assessment of the internal structure of a piece, while imaging can be useful for careful measurement of the geometry of damages and for the evaluation of the restoration performed on the art pieces. However, the technique shows some limitations. Coarse grain stones such as volcanic tuff present relatively large attenuation coefficients^10^, which restricts the penetration depth of the THz radiation, implying that only internal structures near the surface could be detected with this method on those materials. Although this technique is still relatively new and expensive enormous progress is being made by an active community in order to make this technology more economical and user-friendly. We strongly believe that terahertz spectroscopy will become useful tool in the field of art restoration in the near future owing to its non-contact, non-destructive nature.

## Methods

### Terahertz spectroscopy

We used a Menlo Systems fiber coupled terahertz time-domain spectrometer which operates as follows. An Er:fiber mode-locked laser produces 60 fs laser pulses at a central wavelength of 1550 nm. The pulses are delivered by the lasers on two separate fiber ports. One of them is coupled into an optical fiber and guided to a InGaAs photoconductive emitter where terahertz single-cycle transients are produced. The second part of the pulse is sent through an optical delay line and guided using another optical fiber to an InGaAs photoconductive detector. A polyethylene lens is used to collimate the terahertz emission and a silicon beam splitter is used to send ~50% of the terahertz radiation onto the sample after going through a 60 mm focal length polyethylene lens. The terahertz radiation reflected from the sample is transmitted through the beam splitter and a third polyethylene lens is used to focus it down onto the photoconductive detector. By scanning the delay line it is possible to recover the time-dependent waveform of the terahertz transient, this process takes about 2 minutes. The signal recorded contains a first pulse, typically associated to the reflection at the surface of the sample under study, followed by one of several echoes associated to interfaces within the sample that can correspond to inclusions of different stones or other materials or to air-gaps. In order to produce images, the measuring head (emitter, receiver, beam splitter and lenses) are mounted on a pair of motorized translation stages. The system was interfaced in such a way that a rectangular two-dimensional mesh (2 mm step) of positions was traveled by the stages while a time-domain trace was recorded on each position.

### Filling material

We used Jahn M30 as filling agent. It is a common mineral one-component micro backfill grout typically used in conservation of stone objects for the backfilling of hairline cracks with widths from 0.2–5.0 mm. Before applying the grout all dust and loose or deleterious material was removed from the crack in order to facilitate flow and adhesion of the filling material. The stone was pre-wet using a syringe. The backfill grout was mixed with a stirrer at a speed of 3000 rpm for 10 minutes, slowly adding water up to the water content was ultimately 45% of the grout middleweight. After the preparation grout was poured through a sieve and injected to the cavity of the object with a syringe.

## Additional Information

**How to cite this article**: Krügener, K. *et al.* Terahertz meets sculptural and architectural art: Evaluation and conservation of stone objects with T-ray technology. *Sci. Rep.* **5**, 14842; doi: 10.1038/srep14842 (2015).
